# Primary Cancer of the Liver with Glycosuria

**Published:** 1912-10

**Authors:** 


Primary Cancer of the Liver with Glycosuria. Leuret and Creyx, *Jour. de Med. de Bordeaux,* Jan. 28, 1912. A woman aged 63, had had glycosuria with slight azoturia but without polyuria, for four years. She entered their service with copious diarrhoea and intense anæmia. The tentative diagnosis was of pancreatic tumor, though there were no physical signs. At necropsy, a spheroid mass was found in the right lobe of the liver, inclosed in normal hepatic tissue. There was no other cancer and no pulmonary tuberculosis. The exact cause of death seemed to be a gradual exhaustion, there being no acidosis nor other complication. The case was not considered genuinely diabetic. Nothing could be found in the literature bearing on the relation of hepatic tumor to glycosuria nor was there a report of a similar case. (Note.—Some years ago, we saw a case of typic chronic alchoholism with gastritis and moderate contraction of the liver. On necropsy, several discrete hepatic cancers, from the size of a bean to that of a walnut were found. There was no bossilation of the liver and the cancers had, of course, not been diagnosed. There was no other cancerous involvement and there had been no glycosuria. A. L. B.)

